# Notch Signaling between Cerebellar Granule Cell Progenitors

**DOI:** 10.1523/ENEURO.0468-20.2021

**Published:** 2021-05-08

**Authors:** Toma Adachi, Satoshi Miyashita, Mariko Yamashita, Mana Shimoda, Konstantin Okonechnikov, Lukas Chavez, Marcel Kool, Stefan M. Pfister, Takafumi Inoue, Daisuke Kawauchi, Mikio Hoshino

**Affiliations:** 1 Department of Biochemistry and Cellular Biology, National Institute of Neuroscience, National Center of Neurology and Psychiatry, Tokyo 187-8551, Japan; 2 Department of Life Science and Medical Bioscience, School of Advanced Science and Engineering, Waseda University, Tokyo 169-8555, Japan; 3 Department of NCNP Brain Function and Pathology, Graduate School of Medical and Dental Sciences, Tokyo Medical and Dental University, TMDU, Tokyo 113-8510, Japan; 4 Hopp-Children’s Cancer Center Heidelberg (KiTZ), 69120 Heidelberg, Germany; 5 Division of Pediatric Neurooncology, German Cancer Research Center (DKFZ) and German Cancer Consortium (DKTK), 69120 Heidelberg, Germany; 6 Department of Medicine, University of California San Diego, La Jolla, CA 92093; 7 Moores Cancer Center, University of California San Diego, La Jolla, CA 92093; 8 Princess Maxima Center for Pediatric Oncology, 3584 CS Utrecht, The Netherlands; 9 Department of Pediatric Hematology and Oncology, Heidelberg University Hospital, 69120 Heidelberg, Germany

**Keywords:** cerebellar granule cell progenitor, Notch signaling

## Abstract

Cerebellar granule cells (GCs) are cells which comprise over 50% of the neurons in the entire nervous system. GCs enable the cerebellum to properly regulate motor coordination, learning, and consolidation, in addition to cognition, emotion and language. During GC development, maternal GC progenitors (GCPs) divide to produce not only postmitotic GCs but also sister GCPs. However, the molecular machinery for regulating the proportional production of distinct sister cell types from seemingly uniform GCPs is not yet fully understood. Here we report that Notch signaling creates a distinction between GCPs and leads to their proportional differentiation in mice. Among Notch-related molecules, *Notch1*, *Notch2*, *Jag1*, and *Hes1* are prominently expressed in GCPs. *In vivo* monitoring of *Hes1*-promoter activities showed the presence of two types of GCPs, Notch-signaling ON and OFF, in the external granule layer (EGL). Single-cell RNA sequencing (scRNA-seq) and *in silico* analyses indicate that ON-GCPs have more proliferative and immature properties, while OFF-GCPs have opposite characteristics. Overexpression as well as knock-down (KD) experiments using *in vivo* electroporation showed that NOTCH2 and HES1 are involved cell-autonomously to suppress GCP differentiation by inhibiting NEUROD1 expression. In contrast, JAG1-expressing cells non-autonomously upregulated Notch signaling activities via NOTCH2-HES1 in surrounding GCPs, eventually suppressing their differentiation. These findings suggest that Notch signaling results in the proportional generation of two types of cells, immature and differentiating GCPs, which contributes to the well-organized differentiation of GCs.

## Visual Abstract

**Figure:**
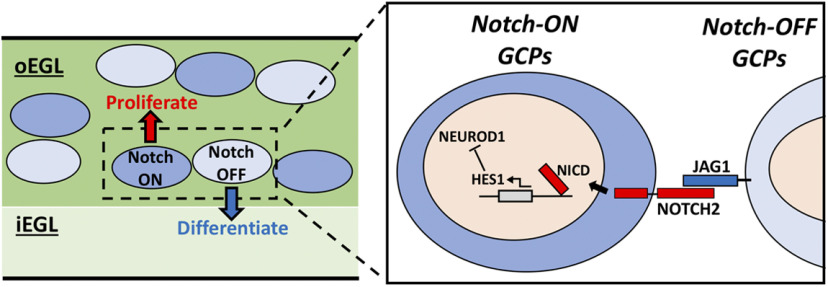

## Significance Statement

This study is the first to succeed in visualization of Notch signaling *in vivo* during cerebellar development. Granule cell progenitors (GCPs) in the outermost layer of the developing cerebellum are a seemingly homogenous cell population, but this study revealed two types of GCPs; more proliferative Notch-ON-GCPs and more differentiative Notch-OFF-GCPs, the latter of which gradually give rise to postmitotic GCs. Our experiments suggest that NOTCH2 and HES1 are involved cell-autonomously to suppress GCP differentiation by inhibiting NEUROD1 expression. In contrast, JAG1-expressing cells non-autonomously upregulated Notch signaling activities via NOTCH2-HES1 in surrounding GCPs, suppressing their differentiation. This study gives new insights into the mechanisms controlling the differences within homogenous cell populations that direct proper and coordinated cell differentiation.

## Introduction

Notch signaling is one of the most important signaling pathways involved in many aspects of life (Tsakonas et al., 1999; Andersson et al., 2011). It is mainly known for the intercellular signaling between “signal-sending cells,” which present the Notch ligand on their cell surfaces and “signal-receiving cells,” which present the Notch receptor (Tsakonas et al., 1999; Andersson et al., 2011). Once the ligand binds to the receptor, the downstream intracellular pathway is activated in the Notch receptor-presenting cells, upregulating expression of *Hes* and *Hey* family transcription factors, and subsequently affecting the binary cell fate of adjacent cells (Tsakonas et al., 1999; Andersson et al., 2011).

Notch signaling molecules were first identified as neurogenic genes in *Drosophila* via systemic genetic screening by the Campos-Ortega group (Lehmann et al., 1983). They identified *Notch*, *δ*, *master mind*, *Enhancer of Split*, which later turned out to constitute a very important signaling pathway, the “Notch signaling pathway.” In *Drosophila* embryogenesis, neuroectodermal cells stochastically differentiate into epidermal cells and neuroblasts in the ratio of 4:1 (Campos-Ortega and Hartenstein, 1985). However, if a neurogenic gene is disrupted, all neuroectodermal cells differentiate into neuroblasts (Lehmann et al., 1983; Tsakonas et al., 1999). This observation led to the notion that neurogenic genes (or Notch signaling) are involved in “lateral inhibition,” by which only a limited number of cells can differentiate into a neural lineage whereby the rest go to an epidermal lineage. Thus, the Notch signaling machinery or lateral inhibition system enables uniform cells to differentiate proportionally into different cell types.

Since then, numerous studies on Notch signaling have identified identical or similar genes in vertebrates, including *Notch1-4*, *Dll1,2,4*, *Jag1,2*, *Maml1,2,3*, *Hes1-7*, and *Hey1,2* (Kageyama et al., 2007; Andersson et al., 2011). Notch signaling is involved in various developmental events in various tissues, including the nervous system not only in invertebrates but also in vertebrates (Louvi and Tsakonas, 2006). For example, Notch signaling is involved in the production of neurons and neural progenitors from radial glia of the mammalian cerebral cortex, precisely regulating the ratio of sister radial glia and sister neuronal cells (Shitamukai et al., 2011). Thus, it is believed that one of the important functions of Notch signaling is to generate different types of cells from uniform cells, which may be the universal basis for the development of multicellular organisms that originated from a single cell, that is, an oocyte or an egg.

The cerebellum contains a tremendous number of granule cells (GCs) that comprise over 50% of the neurons in the entire nervous system (Williams and Herrup, 1988). Numerous GCs enable the cerebellum to properly regulate motor coordination, motor learning, and consolidation, in addition to cognition, emotion and language (Lackey et al., 2018). GC progenitors (GCPs) are mitotic cells located in the outer external GC layer (oEGL) of the developing cerebellum (Chizhikov and Millen, 2003). Although GCPs in the oEGL seem to be uniform, they divide to produce two types of sister cells, GCPs and GCs (Yang et al., 2015). GCs generated from GCPs move to the inner EGL (iEGL) and then migrate radially through the molecular layer (ML) to reach the inner GC layer (IGL; Schilling, 2018). The proportion of sister GCPs and GCs is thought to be precisely regulated at each developmental stage (Miyashita et al., 2017). Disruption of this regulation may cause a smaller cerebellum as observed in cerebellar hypoplasia (Basson and Wingate, 2013). It may also lead to generation of SHH subgroup medulloblastoma, a tumor derived from GCPs (Goodrich et al., 1997).

Several reports have suggested expression of some Notch related molecules in GCPs/GCs (Tanaka et al., 1999; Irvin et al., 2001, 2004; Solecki et al., 2001; Stump et al., 2002; Tanaka and Marunouchi, 2003; Eiraku et al., 2005). The Solecki group showed that overexpression of NOTCH2 and HES1 suppressed the differentiation of GCs as was estimated by neurite length in cultured GCPs/GCs as well as cerebellar explant (Solecki et al., 2001). However, there have been no studies that directly observed Notch signaling between GCPs or that showed the significance of Notch signaling between GCPs for GC development.

In this study, we identified Notch signaling molecules that are abundantly expressed in GCPs. Furthermore, we succeeded in classifying GCPs into Notch-ON and -OFF cells, the former of which are more immature and proliferative, whereas the latter are less proliferative and more differentiated. Intercellular interaction of JAG1 and NOTCH2 increase HES1 expression in NOTCH2-expressing cells, which eventually drive the cells to be Notch-ON cells. NOTCH2 and HES1 are autonomously involved in keeping GCPs in a proliferative state, while JAG1-expressing cells had similar effects non-autonomously. We also found that HES1 suppresses the expression of NEUROD1, an important factor that promotes GC differentiation, suggesting that Notch-signaling causes GCPs to remain in a proliferative state by suppressing NEUROD1 expression. This study reveals a new function of Notch signaling in GC development and provides insights into the machinery underlying how different cell types are proportionally generated from uniform cells.

## Materials and Methods

### Animals

All mouse experiments were approved by the Animal Care and Use Committee of the National Institute of Neuroscience, Japan. Mice were housed in SPF conditions and maintained on a 12/12 h light/dark cycle with free access to food and water. ICR pups were obtained from SLC. In all analyses, we used pups without identifying the sex of each animal.

### Quantitative PCR (qPCR)

GCP isolation and qPCR was performed as previously described (Kutscher et al., 2020). In brief, 5 million GCPs were plated per well in a six-well plate and cultured for 48 h with or without 200 mm smoothened agonist (SAG, Merck). GCPs were harvested and cDNA was generated with Superscript IIkit (Invitrogen). Relative gene expression was compared with the geometric mean of *Hrpt1*, *Rpl27*, and *Rer1* (Thomas et al., 2014). The primer sequences used are as following:

*Hrpt1*: CAAACTTTGCTTTCCCTGGT and TCTGGCCTGTATCCAACACTTC,

*Rpl27*: AAGCCGTCATCGTGAAGAACA and CTTGATCTTGGATCGCTTGGC,

*Rer1*: GCCTTGGGAATTTACCACCT and CTTCGAATGAAGGGACGAAA,

*Ccnd2*: GAGAAGCTGCCCTGATCCGCA and CTTCCAGTTGCAATCATCATCGACG,

*Notch1*: GCTGCCTCTTTGATGGCTTCGA and CACATTCGGCACTGTTACAGCC,

*Notch2*: CCACCTGCAATGACTTCATCGG and TCGATGCAGGTGCCTCCATTCT,

*Notch3*: GGTAGTCACTGTGAACACGAGG and CAACTGTCACCAGCATAGCCAG,

*Notch4*: GGAGATGTGGATGAGTGTCTGG and TGGCTCTGACAGAGGTCCATCT,

*Hes1*: GGAAATGACTGTGAAGCACCTCC and GAAGCGGGTCACCTCGTTCATG,

*Hes5*: CCGTCAGCTACCTGAAACACAG and GGTCAGGAACTGTACCGCCTC,

*Jag1*: TGCGTGGTCAATGGAGACTCCT and TCGCACCGATACCAGTTGTCTC,

*Jag2*: CGCTGCTATGACCTGGTCAATG and TGTAGGCGTCACACTGGAACTC,

*Dll1*: GCTGGAAGTAGATGAGTGTGCTC and CACAGACCTTGCCATAGAAGCC,

*Dll3*: CCAGCACTGGATGCCTTTTACC and ACCTCACATCGAAGCCCGTAGA,

*Dll4*: GGGTCCAGTTATGCCTGCGAAT and TTCGGCTTGGACCTCTGTTCAG.

### Plasmids

Expression vectors of HES1, 5, JAG1, and NEUROD1, and sh vectors for *Notch1*, *Notch2*, *Hes1*, *Hes5*, *Jag1*, and *NeuroD1* were constructed as previously reported (Kawauchi et al., 2006). All cloned expression fragments were inserted into a pCAGGS vector (GE Healthcare). The primers sequences, used for cloning are, for HES1: 5′-ATGCCAGCTGATATAATGG-3′ and 5′-TCATCCTCTGGTCCGCT-3′, HES5: 5′-ATGGCCCCAAGTACCGT-3′ and 5′-TCATCCTCTGGTCCGCT-3′, JAG1: 5′-TCCACGGAGTATATTAGAGCC-3′, 5′-GCTAGCACACTCATCGATG-3′, 5′-AACCCCTGCTTGAATGGG-3′and 5′-CTATACGATGTATTCCATCCGGTT-3′, and NEUROD1: 5′-ATGACCAAATCATACAGCGA-3′ and 5′-CTAATCGTGAAAGATGGCAT-3′. sh vectors were generated by inserting the double-stranded oligonucleotides into a mU6 pro vector. The targeting sequence for each vector was designed by siDirect 2.0 (Naito et al., 2009). Sequences are, *Notch1*: #1 5′-AAGGTGTATACTGTGAAATCAAC-3′, #2 5′-CTGTAACAGTGCCGAATGTGAGT-3′, *Notch2*: #1 5′- AGGCCTTAATTGTGAAATTAATT-3′, #2 5′-GAGGTGATAGGCTCTAAGATATT-3′, *Hes1*: #1 5′-GAGGCGAAGGGCAAGAATAAATG-3′, #2 5′-TTGGATGCACTTAAGAAAGATAG-3′, *Hes5*: #1 5′-CCGCATCAACAGCAGCATAGAGC-3′, #2 5′-CCGTCAGCTACCTGAAACACAGC-3′, *NeuroD1*: #1 5′-GCCTAGAACGTTTTAAATTAAGG-3′, #2 5′-TGGCAACTTCTCTTTCAAACACG-3′. pCAG-H2BGFP vectors and pCAG-mCherry were a gift from N. Masuyama. p*Hes1*-d2GFP, p*Hes5*-d2GFP and *Hes1*p-venus vectors were gifts from R. Kageyama (Kohyama et al., 2005; Ohtsuka et al., 2006). pCAG-H2B-BFP (pTagBFP-H2B) was purchased from evrogen (catalog #FP176). Coding region of *Hes1* or *Hes5* was inserted into a pEGFP-N3 vector to generate HES1-fusion-GFP and HES5-fusion-GFP vectors.

### Antibodies and immunohistochemistry

Detailed protocols for immunohistochemistry were described previously (Seto et al., 2014). Briefly, neonatal mice were fixed with 4% PFA and embedded with O.C.T compound (Sakura Finetek). Frozen brains were sagittally sectioned into 18-μm slices with a cryostat (CM3050 S; Leica). Cryosections were incubated at room temperature with 1% normal donkey serum containing 0.2% PBST (blocking solution) for 1 h. After blocking, sections were incubated with primary antibodies diluted with blocking solutions at 4° for 16 h. The following primary antibodies were used, goat anti-Notch1 (1:500; sc-6015; Santa Cruz Biotechnology), goat anti-Notch2 (1:500; sc-7423; Santa Cruz Biotechnology), rabbit anti-Jagged1 (1:500; ab7771; Abcam), chicken anti-GFP (1:1000; GFP-1010; Aves), rabbit anti-RFP (1:500; PM005; MBL; RFP antibody was used to enhance CAG-mCherry signals), rabbit anti-Atoh1 (1:200; Yamada et al., 2014), rat anti-Ki67 (1:500; 14-5698-82; eBioscience), rabbit anti-Pax6 (1:500; PRB-278P; BioLegend), and goat anti-NeuroD (N-19; 1:200; sc-1084; Santa Cruz Biotechnology). Subsequently, slides were rinsed with PBS and incubated with secondary antibodies conjugated with Alexa Fluor 488, Alexa Fluor 568, or Alexa Fluor 647 (1:400; Abcam) and DAPI (25 μg/ml; Invitrogen) in blocking buffer in PBS at room temperature for 2 h. Slides were rinsed with PBS again and mounted with Permafluor.

### *In vivo* electroporation

*in vivo* electroporation to neonatal mice was described previously (Owa et al., 2018). Expression plasmids were diluted to 1 μg/μl, shRNAs to 2 μg/μl and fluorescent protein vectors to 0.5 μg/μl in milliQ. p*Hes1*-d2GFP, p*Hes5*-d2GFP and *Hes1*p-venus vectors were diluted to 1 μg/μl in milliQ. Fastgreen was added to visualize the plasmid solution. Pups were anesthetized on ice before electroporation; 10-μl plasmid solutions were injected into P5 or P6 ICR cerebella over the skull. Electric pulses (50 ms in duration, 80 V, seven times) were delivered to mice with 150-ms interval using forceps-type electrodes (NEPA gene). The pups were kept warm at 37° until recovered and returned to the litter. Pups were fixed a few days after electroporation as indicated. For “double electroporation” experiments (Fig. 9*A–C*), pups were temporarily returned to the litter after the first electroporation and the procedure was repeated 6 h later. In Figure 9*D–F*, EdU (10 mg/ml) diluted in PBS (50 mg/kg, 20 μl total volume) was introduced by intraperitoneal injection immediately following electroporation.

### Slice culture and time lapse imaging

Cerebellar slice culture was prepared as previously reported (Owa et al., 2018). Electroporated postnatal day (P)6 or P7 mouse cerebella were sliced into 250-μm sagittal sections with a vibratome. Slices were cultured at 37° for 6 h, and time lapse imaging was taken with a confocal microscope, FV3000 (Olympus). Pictures were taken every 5 min. In Figure 2*D-F*, 1 μl DMSO or γ secretase inhibitor RO4929097 (5 nm, Selleck) were added to the medium just before the imaging.

### Image acquisition and quantification

All images were acquired from midline vermis region of Lobules IV–VI with confocal microscopy, LSM780 (Carl Zeiss) and FV3000 (Olympus). Acquired images were analyzed with ImageJ (RSB). The analyses to calculate the rate of undifferentiated states of GCPs were done by using the “Cell counter in Plugins” in ImageJ. All of the GFP (or mCherry)-positive cells in EGL, ML, and IGL were included in the analyses. The analyses to quantify the protein level of GFP and mCherry in Figure 3, and NEUROD1 in Figure 10, were done by using “Measures in Analyze” in ImageJ. The NEUROD1 brightness of GFP negative cells located in the next to GFP-positive cells were used as control.

### Cell culture and transfection

Cell culture and transfection were performed as previously reported (Miyashita et al., 2020). Neuro2a (N2a) cells were cultured in DMEM containing 10% FBS and 100 U/ml penicillin-streptomycin. Five million cells were plated per well in a six-well plate and 24 h after the passage, transfection was performed with transfectin reagent (Bio-Rad); 1 μg expression vector plasmid DNA and 2 μg sh plasmid DNA were introduced in each well.

### Western blotting

N2a cells were harvested 48 h after the transfection. Detailed protocols of western blotting were described previously (Shiraishi et al., 2019). Membranes were incubated with primary antibodies at 4° overnight. The following primary antibodies were used, rabbit anti-β-actin (1:1000; MBL), rabbit anti-GFP (1:2000; MBL-598; MBL), and rabbit anti-Jagged1 (1:1000; ab7771; Abcam). After membranes were incubated with secondary antibodies at room temperature for 1 h, HRP substrate (Millipore) was added and immuno-signals were detected by LAS4000 (Fujifilm). In quantification, all of the targeted proteins expression levels are normalized by the expression level of β-actin.

### Smart-Seq [single-cell RNA sequencing (scRNA-seq)]

Cells were prepared from P7 cerebella using Percoll density gradient centrifugation as described previously (Kawauchi et al., 2012) followed by capturing the cells using the C1 Fluidigm systems with 96-well chips. The total RNAs were extracted from single sorted cells and the library was prepared for the subsequent RNAseq.

### Data processing for scRNA-seq data

The sequencing reads were aligned to mm10 reference using STAR 2.4.1d (Dobin et al., 2013). Gene expression counts per cell were computed with HTseq-count tool 0.6.1 (Anders et al., 2015). Quality control for the alignments was performed with Qualimap v2.2.1 (Okonechnikov et al., 2016). The computed counts were further processed with Seurat v3 as described (Stuart et al., 2019). All cells belonging to the cluster 0 (Fig. 4*D*,*F*) were categorized by *Notch2* expression to *Notch2*-positive-cells (*Notch2 *>* *0) and *Notch2*-negative-cells (*Notch2 *=* *0). Differentially expressed genes (DEG) between *Notch2*-positive-cells and *Notch2*-negative cells were determined with DESeq2 (Love et al., 2014). Genes with adjusted *p* < 0.01 were defined as DEGs (582 upregulated, 74 downregulated).

Unchanged genes were selected from genes with adjusted *p* > 0.05. GSEA analysis was performed with the identified DEGs against NOTCH-REACTOME dataset (Molecular Signatures Database v7.1, M10189, Signaling by NOTCH). DAVID was used to conduct the Gene Ontology (GO) analysis (https://david.ncifcrf.gov).

### Data availability statement

scRNA-seq data used in this study were available in Gene Expression Omnibus (GSE153313).

### Code availability

All the codes used in this study are available from the lead contact (hoshino@ncnp.go.jp).

### Statistical analyses

Individual animals or trials are regarded as biological replicates. All controls were prepared under the same experimental conditions. All data are presented as mean ± SEM. Statistical tests were performed by Student’s *t* test and one-way ANOVA with Bonferroni’s *post hoc* test; *p* values were represented as; N.S. for *p* > 0.05; *p < 0.05, **p < 0.01, ***p < 0.001.

## Results

### Expression of Notch-related molecules in cerebellar GCPs

**Figure 1.**
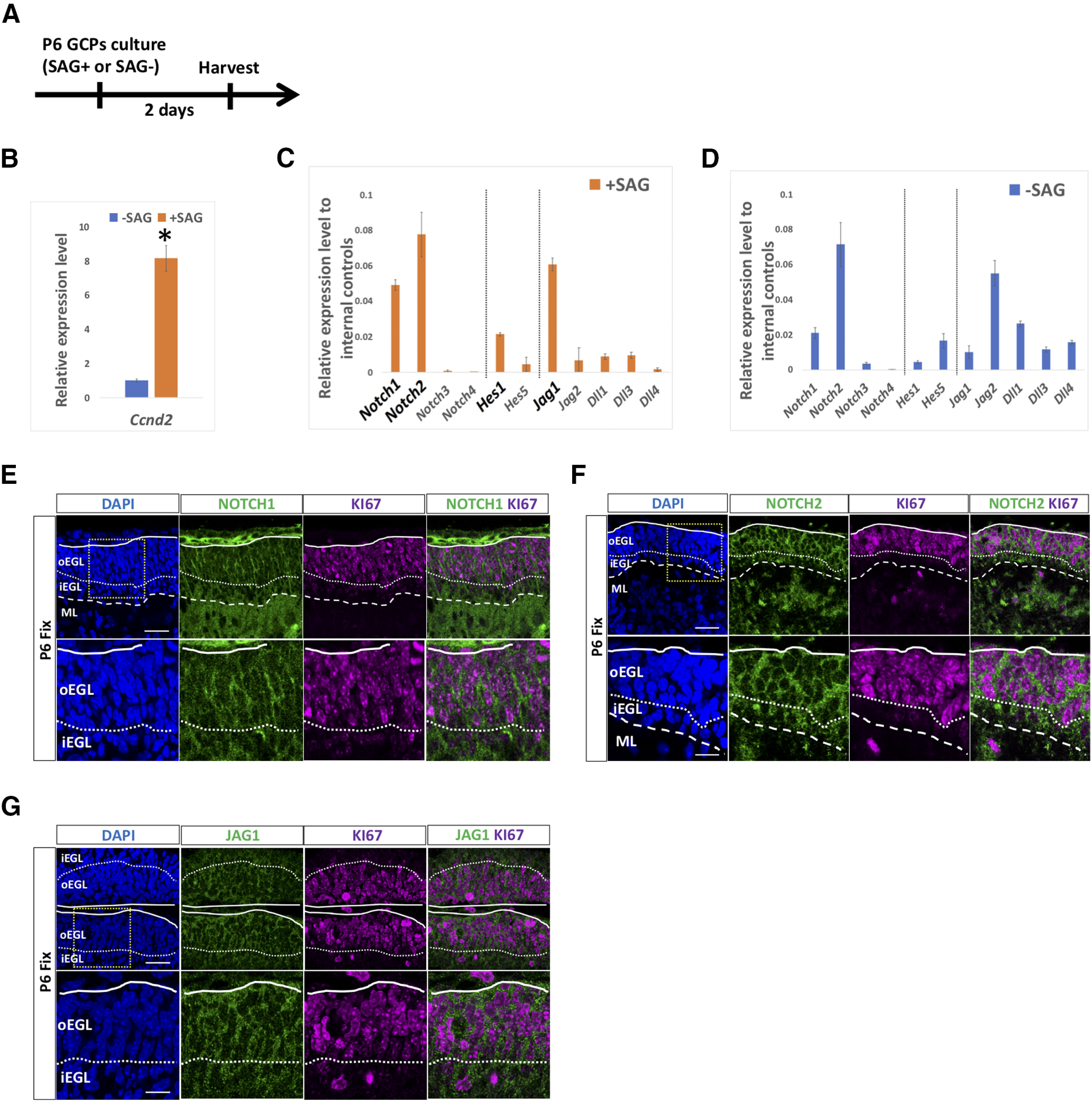
Expression of Notch signaling molecules in cerebellar GCPs. ***A–D***, Gene expression of Notch receptors (*Notch1,2,3,4*), Hes family genes (*Hes1,5*), and ligands (*Jag1,2, Dll1,3,4*) was estimated by quantitative RT-PCR using GCP-like (SAG+) or GC-like (SAG–) cells that were purified from P6 cerebella (***A***). *Hrpt1*, *Rpl27*, and *Rer1* were used as internal controls (***C***, ***D***). *Ccnd2* expression is a marker of GCPs used to monitor the culture conditions (***B***). Data are shown as mean ± SEM; *p < 0.05, Student’s *t* test. ***E–G***, Immunostaining with indicated antibodies on sagittal cerebellar sections. Sections were co-stained with DAPI, a nuclear marker. Within the EGL, nearly all Ki67-positive cells are GCPs (Chizhikov and Millen, 2003; Yang et al., 2015; Schilling, 2018). Lobule IV/V or VI is shown. Differences were not observed as to their expression along an anterior-posterior axis. Scale bars: 30 and 15 μm (***E–G***).

Previously, several studies reported that Notch signaling-related molecules were expressed in the cerebellar EGL (Tanaka et al., 1999; Irvin et al., 2001, 2004; Solecki et al., 2001; Stump et al., 2002; Tanaka and Marunouchi, 2003; Eiraku et al., 2005). However, in all cases, no discrimination was made between the expression in GCPs and GCs. Therefore, we isolated GCPs from cerebella at P6 according to our method (Kutscher et al., 2020) and cultured them for 2 d with or without SAG, an activator of SHH signaling. Under this condition, SAG-treated and non-treated cells possess characteristics very similar to those of GCPs and GCs, respectively (Kutscher et al., 2020). Consistently, *Ccnd2*, known as a mitotic GCP marker, were expressed highly in SAG-treated cells, and expressed very low in non-treated cells (Fig. 1*A*,*B*). We performed quantitative RT-PCR (qPCR) to determine the expression of Notch signaling-related genes. In SAG-treated cells, or GCP-like cells, *Notch1*, *Notch2*, *Hes1*, and *Jag1* were all prominently expressed while expression of *Notch3*, *Notch4*, *Hes5*, *Jag2*, *Dll1*, *Dll3*, and *Dll4* were low (Fig. 1*C*). In SAG-non-treated cells, we observed considerable expression of *Notch1*, *Notch2*, *Hes5*, and *Jag2* (Fig. 1*D*). Because our aim was to investigate the importance of Notch signaling among GCPs in the EGL, we focused on *Notch1*, *Notch2*, *Hes1*, and *Jag1* in this study. Next, we performed immunostaining with antibodies against NOTCH1, NOTCH2, and JAG1 on P6 cerebellum along with KI67, a marker for mitotic cells. NOTCH1, NOTCH2, and JAG1, exhibited honeycomb-like staining signals in the oEGL and iEGL (Fig. 1*E–G*), suggesting that those membrane proteins were expressed by GCPs and GCs in the developing cerebellum.

### Notch2-Hes1 signaling is active in the EGL

**Figure 2.**
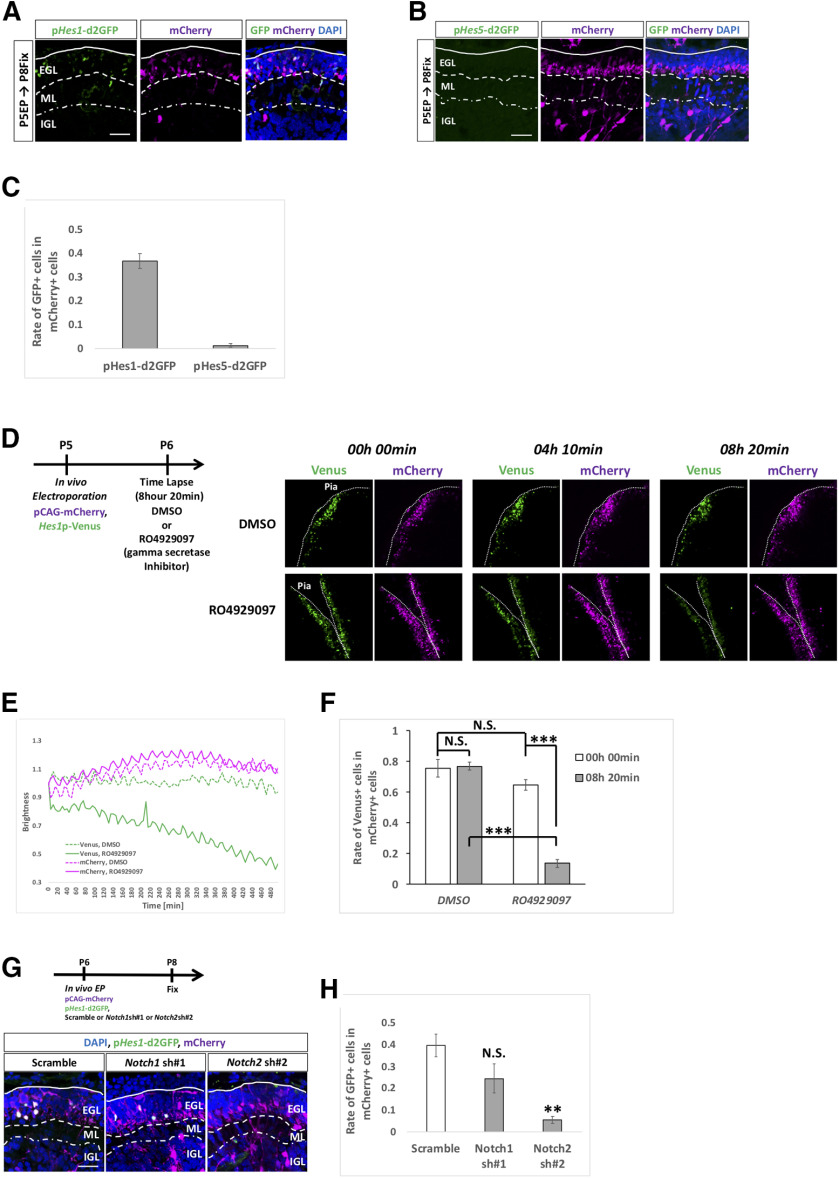
Hes1 promoter activity in EGL is reduced by a γ secretase inhibitor and Notch2 KD. ***A–C***, To check the activities of *Hes1* and *Hes5* promoters, p*Hes1*-d2GFP (***A***) and p*Hes5*-d2GFP (***B***) were electroporated into P5 EGL with pCAG-mCherry vector. In the EGL, only *Hes1* promoter activities were detected (***A***–***C***). Rates of GFP-positive cells in mCherry+ cells were calculated (***C***). Animal numbers: *N* = 7 for p*Hes1*-d2GFP and *N* = 3 for p*Hes5*-d2GFP. Scale bars: 30 μm (***A***, ***B***). ***D–F***, p*Hes1*-Venus and pCAG-mCherry were electroporated to P5 mouse, followed by slice culture at P6 (***D***). Time lapse images were taken for 8 h 20 min in the presence of RO4929097 (γ secretase inhibitor) or control DMSO that were administered at the start of the time lapse. Decline of the Venus brightness and rate of the Venus-positive cells in mCherry+ cells were observed only in the RO4929097 treated slices (***D–F***). Scale bars: 80 μm (***A***). Data are shown as mean ± SEM; ***p < 0.001, one-way ANOVA with Bonferroni’s *post hoc* test. Animal numbers: *N* = 3 for DMSO and RO4929097. ***G***, ***H***, p*Hes1*-d2GFP and pCAG-mCherry were electroporated with indicated KD vectors to P6 cerebella, followed by fixation at P8. KD for *Notch2* but not for *Notch1* reduced the rates of GFP+ cells in mCherry+ cells. Animal numbers: *N* = 4 for *Notch1* sh#1 and *Notch2* sh#2. Scale bars: 30 μm (***F***). Data are shown as mean ± SEM; **p < 0.01, Student’s *t* test.

Given that some Notch-related molecules were expressed in GCPs in the developing cerebellum, we tried to monitor Notch signaling activities in GCPs in the EGL. To this end, we used vectors, p*Hes1*-d2GFP and p*Hes5*-d2GFP, which were designed to express short half-life GFP (d2GFP) under the control of *Hes1* and *Hes5* promoters, respectively (Ohtsuka et al., 2006). We performed *in vivo* electroporation (Owa et al., 2018; Chang et al., 2019) with p*Hes1*-d2GFP or p*Hes5*-d2GFP plus an mCherry-expressing vector (pCAG-mCherry) on GCPs in the EGL at P5 and fixed electroporated cerebella 3 d after electroporation. While p*Hes1*-d2GFP gave rise to significant signals in the EGL, signals for p*Hes5*-d2GFP were barely observed (Fig. 2*A–C*). This discrepancy in the promoter activities of *Hes1* and *Hes5* in the EGL is consistent with that of the levels of expression strength of *Hes1* and *Hes5* in SAG-treated cells (Fig. 1*C*).

We next tried to confirm whether this *Hes1* promoter activity in the EGL is under the control of Notch signaling. For this purpose, we performed time lapse observation of cerebellar slices containing a *Hes1* promoter monitoring vector in the presence or absence of RO4929097. RO4929097 is a specific γ secretase inhibitor that prevents the cleavage of Notch intracellular domain (NICD), leading to inhibition of its downstream signaling cascades (Luistro et al., 2009). Of note, we used p*Hes1*-Venus instead of p*Hes1*-d2GFP because of requirement of stronger fluorescence signals during time-lapse recordings (Kohyama et al., 2005). We electroporated p*Hes1*-Venus plus pCAG-mCherry to P5 cerebella, generated cerebellar slices at P6 and performed time lapse observations in the presence or absence of RO4929097 for >8 h (Fig. 2*D–F*). The kinetics of the fluorescence intensities clearly showed that RO4929097 gradually but dramatically decreased the Venus intensity reflecting *Hes1* promoter activity, while the Venus intensity was not changed in control (DMSO application) slices during the recordings (Fig. 2*D*,*E*). Also, the rate of Venus-positive cells in mCherry+ cells were dramatically decreased only in the RO4929097 applied slices (Fig. 2*D*,*F*).

These results suggest that fluorescence signals for the *Hes1* promoter in the EGL are regulated by Notch signaling.

Among Notch family genes, *Notch1* and *Notch2* were strongly expressed in GCP-like cells (Fig. 1*C*) and in the EGL (Fig. 1*E*,*F*). Therefore, we aimed to identify which Notch gene is responsible for the *Hes1* promoter activity in the EGL. For this purpose, we electroporated short hairpin (sh)-RNAs for *Notch1* or *Notch2* plus p*Hes1*-d2GFP and pCAG-mCherry into P6 cerebella and fixed them at P8 (Fig. 2*G*). Counting GFP-positive cells in mCherry+ cells showed that knock down (KD) of *Notch2* significantly downregulated the *Hes1* promoter activity, while KD of *Notch1* did not (Fig. 2*G*,*H*), suggesting that Notch2-Hes1 signaling is active in the EGL during cerebellar development.

### Notch signaling-ON and signaling-OFF GCPs in the oEGL

**Figure 3.**
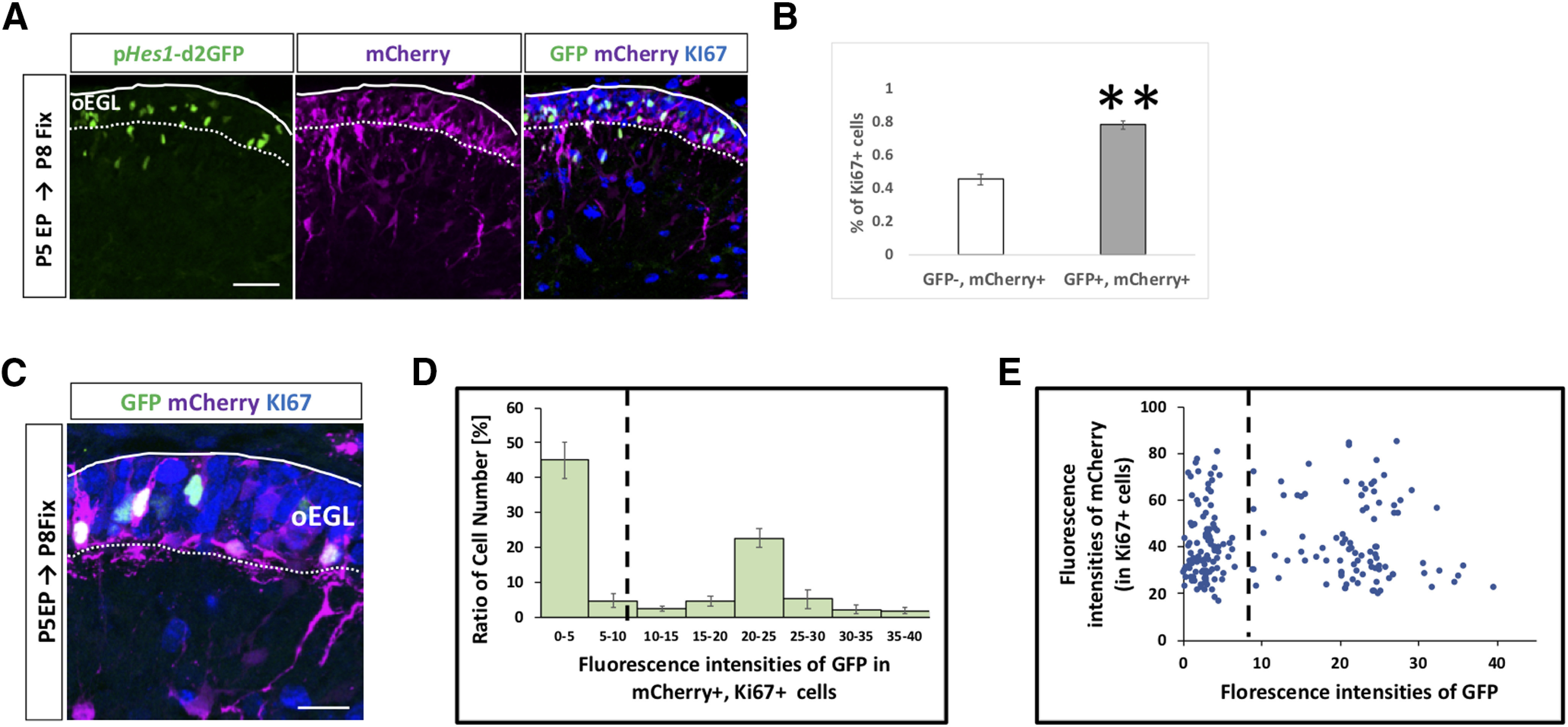
GCPs are divided into two subgroups Notch signaling ON and OFF cells *in vivo*. ***A–E***, *Hes1* promoter activity was stronger in GCPs than GCs (***A***, ***B***). Heterogeneity was observed in *Hes1* promoter activities among (KI67-positive) GCPs in the oEGL (***C–E***). The dashed line represents the threshold of intensity visible by eye (***D***, ***E***). Animal numbers: *N* = 3 for the analysis of ***B*** and *N* = 7 for ***D,E***. Scale bars: 45 μm (***A***) and 20 μm (***C***). Data are shown as mean ± SEM; **p < 0.01, Student’s *t* test.

We electroporated with p*Hes1*-d2GFP and pCAG-mCherry into P5 EGL and immunostained cerebellar samples at P8 with KI67. Interestingly, among electroporated (mCherry-expressing) cells, GFP-positive cells were more mitotic (KI67-positive) than GFP-negative cells (Fig. 3*A*,*B*). This suggests that *Hes1*-promoter active cells tend to remain as proliferating GCPs in the oEGL compared with the negative cells for that activity. In the same experiment, we quantified the fluorescence intensity of GFP in each GCP (KI67-positive cell) in the oEGL (Fig. 3*C–E*). Surprisingly, the fluorescence intensities for GFP exhibited a clear bimodal distribution (Fig. 3*D*,*E*), while those for control mCherry appeared unimodal (Fig. 3*E*). This suggests that GCPs in the oEGL comprise two types of cells, ON and OFF cells, for Notch2-Hes1 signaling.

**Figure 4.**
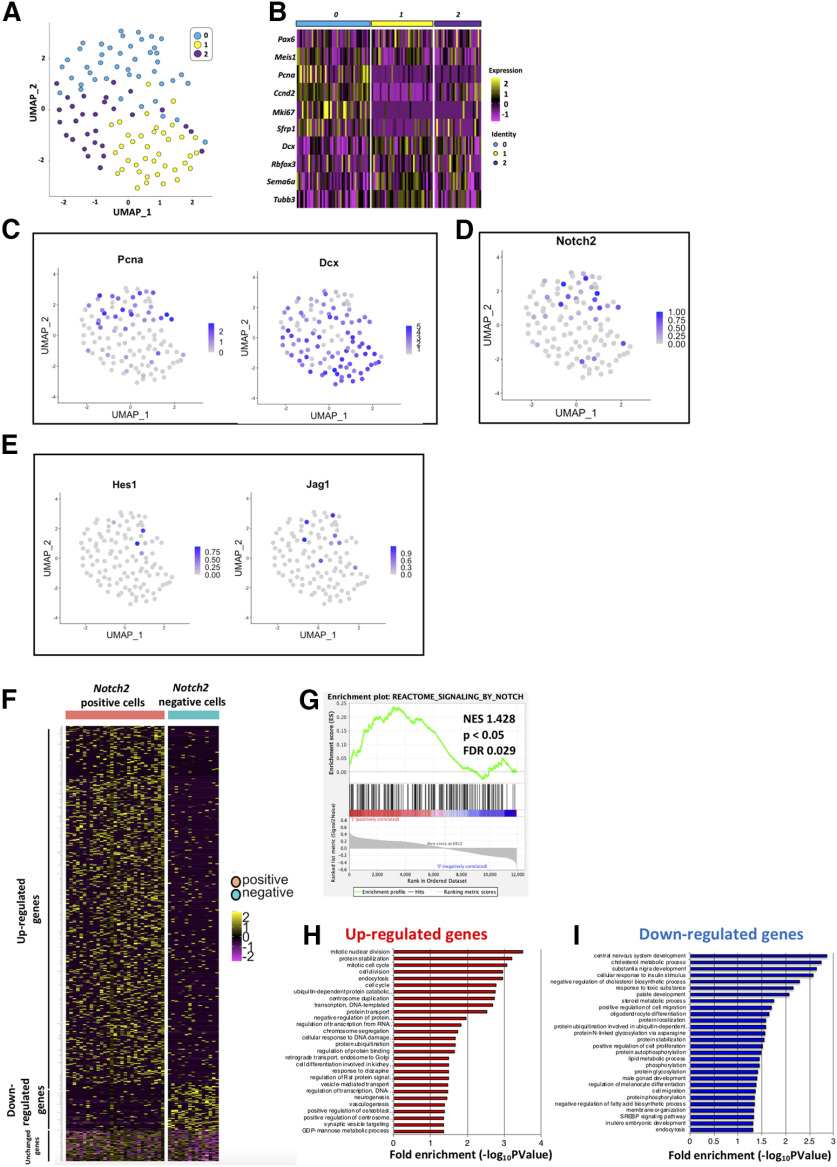
GCPs are divided into two subgroups Notch signaling ON and OFF cells *in silico.* ***A***, scRNA-seq (Smart-seq) analysis of 109 GCPs purified from P7 mice. Uniform manifold approximation and projection (UMAP) dimensional reduction were performed and three clusters were identified (0,1,2). ***B***, Heatmap for expression of GC-lineage markers (*Pax6*, *Meis1*), GCP markers (*Pcna*, *Ccnd2*, *Mki67*, *Sfrp1*), and GC markers (*Dcx*, *Rbfox3*, *Sema6a*, *Tubb3*) in cells of distinct clusters. Molecular features of distinct clusters suggested that cluster 0 corresponded to GCPs and cluster1 and 2 to GCs. ***C–E***, Normalized expression of selected genes are visualized onto the UMAP-dimension (***C***). *Pcna* (a marker for GCPs) are mostly expressed in Cluster 0, while *Dcx* (a marker for GCs) is expressed in Cluster1,2. *Notch2*, *Hes1*, and *Jag1* expressions are also shown (***D***, ***E***). ***F***, GCPs, which were extracted from scRNA-seq of P7 mice (Cluster 0 cells), were grouped by *Notch2* expression (positive and negative cells, please also see Materials and Methods). Expression profiles of 656 DEG between *Notch2*-positive and *Notch2*-negative GCPs were visualized by the heatmap. Expression of 582 genes were upregulated in *Notch2*-positive GCPs, while that of 74 genes were downregulated. ***G***, GSEA of upregulated genes versus downregulated genes in Notch2-positive cells was performed with the REACTOME_SIGNALING_BY_NOTCH dataset. NES, normalized enrichment score; FDR, false discovery rate. ***H***, ***I***, GO analysis was performed for the 582 upregulated genes in *Notch2*-positive GCPs (***H***) and for 74 downregulated genes (***I***).

Next, we performed scRNA-seq on primary GCPs collected from P7 cerebella using the Smart-seq technology. From six independent batches, total 109 cells were successfully subjected to RNA-seq. We performed dimensional compression of the obtained gene expression data into a two-dimensional matrix employing the Seurat software and identified three clusters of single cells (Fig. 4*A*). From the result, it was revealed that GCP and GC markers were highly expressed in the cluster 0 and 1/2 cells, respectively (Fig. 4*B*,*C*). As we found that Notch signaling in GCPs is dependent on *Notch2* (Fig. 2*G*,*H*), we investigated *Notch2* expression in the single cells. In the GCP cluster (cluster 0), 29 cells were *Notch2*-positive, while 15 were negative (Fig. 4*D*). Although the expression of *Jag1* and *Hes1* was detected by quantitative RT-PCR in GCPs (Fig. 1*B*), their expression was found only in limited number of cells in this scRNA-seq analyses, probably because of the limitation for detecting their transcripts under this experimental condition (Fig. 4*E*). We further classified the GCPs cluster into two groups, *Notch2*-positive and *Notch2*-negative cells, which showed dramatically distinct expression profiles (Fig. 4*F*).

In the differential expression analysis using the DESeq2 software (Love et al., 2014), we identified 656 DEG between *Notch2*-positive and *Notch2*-negative GCPs (Fig. 4*F*). Expression of 582 genes was upregulated in *Notch2*-positive GCPs, while that of 74 genes was downregulated (Fig. 4*F*). This clearly suggests that these two populations, *Notch2*-positive and *Notch2*-negative GCPs have quite different molecular characteristics. We performed gene set enrichment analysis (GSEA) to test for a potential enrichment of Notch signaling in *Notch2-*positive and *Notch2*-negative GCPs by comparing 656 DEG to the “REACTOME_SIGNALING_BY_NOTCH” gene set in the Molecular Signatures Database v7.1 (M10189, Signaling by NOTCH). As a result, we found that highly expressed genes in *Notch2*-positive/negative GCPs in our data match to the upregulated/downregulated genes in Notch signaling ON cells, significantly (Fig. 4*G*). We then performed GO analysis for these DEGs (Fig. 4*H*,*I*). Several of the significant GO categories for upregulated genes in *Notch2*-positive GCPs were related to cell cycle progression (Fig. 4*H*), while some GOs for upregulated genes in *Notch2* negative GCPs implicated cell differentiation events, including cell migration (Fig. 4*I*). These findings suggest that Notch-signaling ON GCPs are a more proliferative and less differentiated population, while Notch signaling-OFF GCPs are less proliferative and more differentiated.

### Notch2-Hes1-dependent Notch signaling maintains GCPs in immature and proliferative state

**Figure 5.**
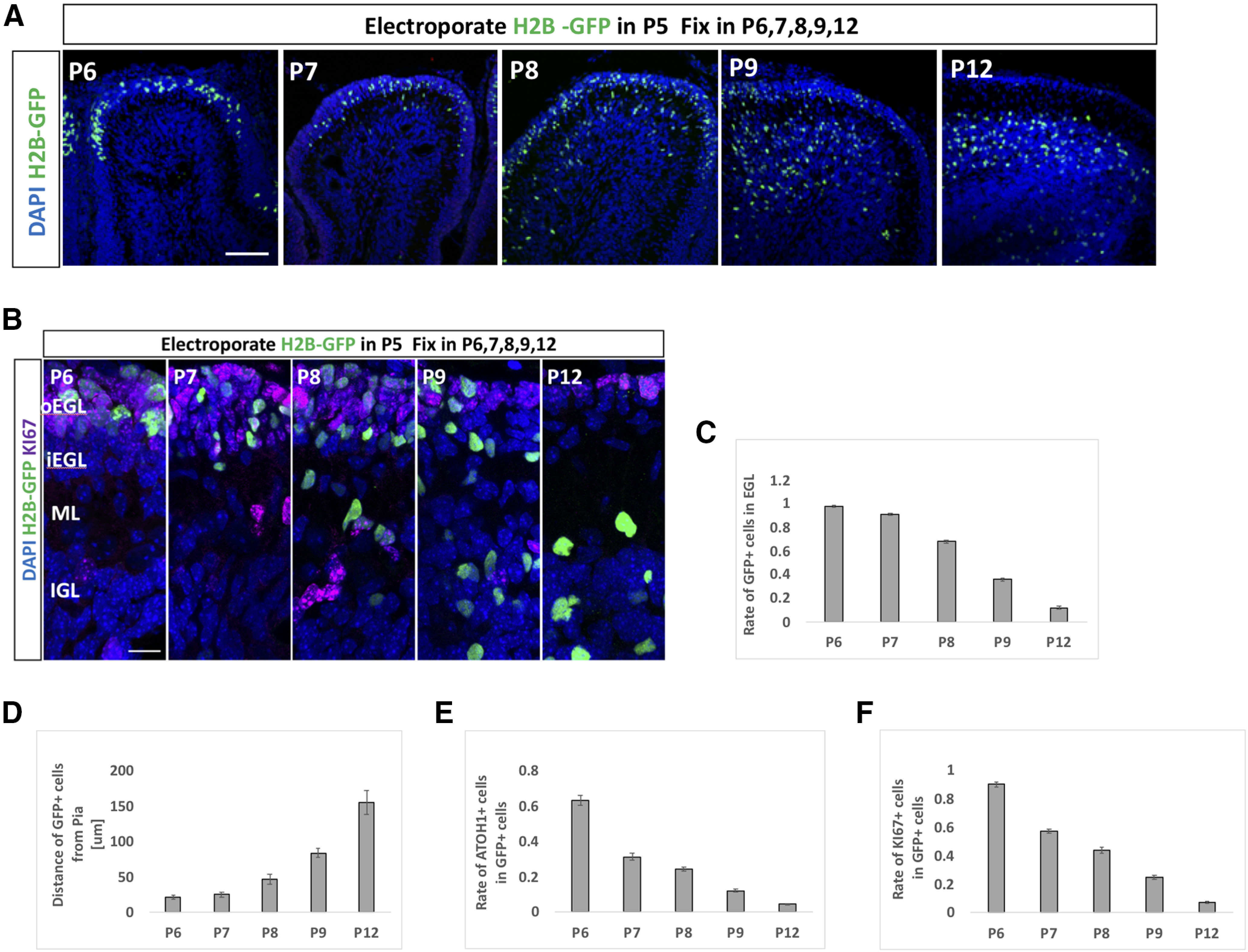
Dynamics of electroporated cells in cerebellar development. ***A–F***, pCAG-H2B-GFP was electroporated to P5 mouse EGL. Electroporated mice were fixed at P6, P7, P8, P9, or P12 and subjected to further analyses (***A***, ***B***). Since GC-lineage cells migrate from EGL to IGL during their maturation (Schilling, 2018), we examined the rate of transfected cells in the EGL and distance of the transfected cell position from the pial matter (***C***, ***D***). We also checked the rates of ATOH1-positive (***E***) and KI67-positive (***F***) cells in GFP-positive cells. Because plasmids were mainly introduced to Lobule IV/V or VI by electroporation in our experimental condition, all analyses in this study were conducted in Lobule IV/V or VI. Scale bars: 80 μm (***A***) and 15 μm (***B***).

We and others previously developed an *in vivo* electroporation gene transfer method for GCPs during cerebellar development (Owa et al., 2018; Chang et al., 2019). In this study, we further characterized the dynamics of differentiation and migration processes of electroporated cells (Fig. 5). The EGL was electroporated with a nuclear-localizing GFP (pCAG-H2B-GFP) at P5 and fixed at P6, P7, P8, P9, and P12 (Fig. 5*A*,*B*). By this method, GCPs facing the cerebellar surface, or the pia mater, were transfected with the expression vector and then examined as they migrated inwardly as development proceeds (Fig. 5*A–D*). The rate of ATOH1 or KI67-positive cells in electroporated cells were gradually decreased (Fig. 5*E*,*F*), reflecting gradual differentiation from GCPs to GCs.

**Figure 6.**
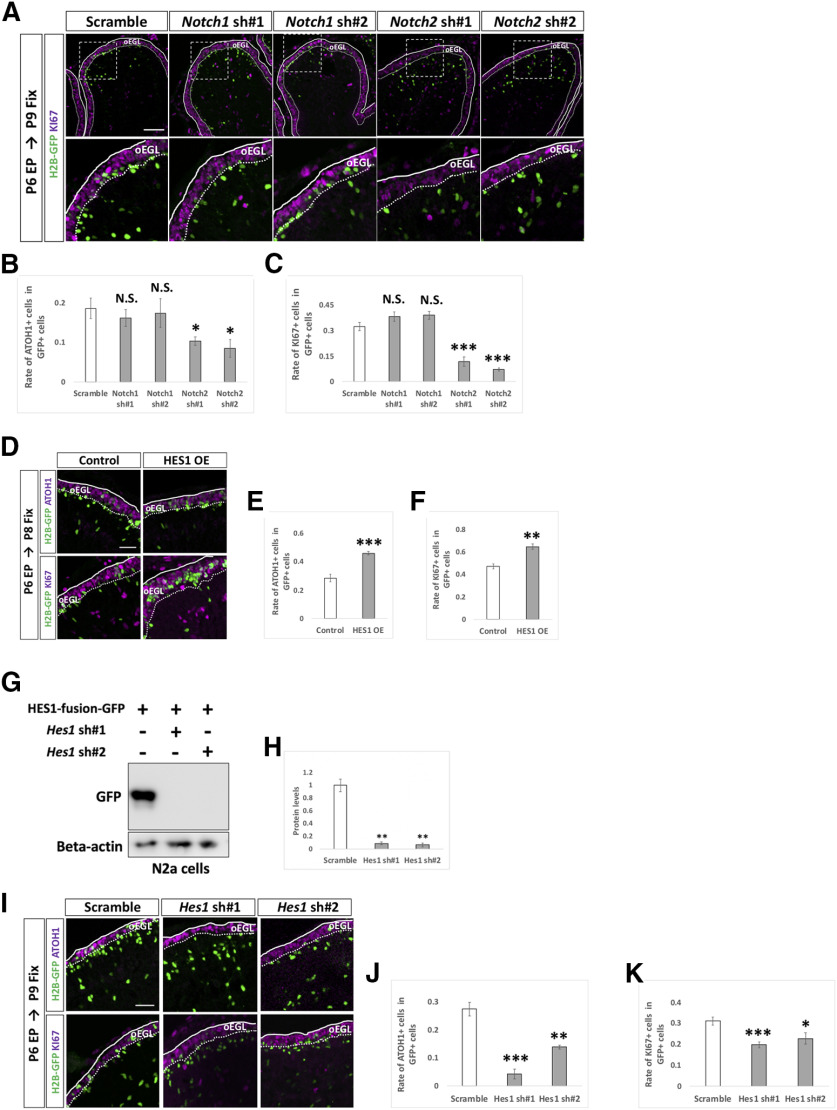
NOTCH2 and HES1 tend to maintain GCPs in immature and proliferative state. ***A–C***, Electroporation of *Notch1,2 kDa* vectors plus pCAG-H2B-GFP into the EGL (***A***). Differentiation of electroporated cells were evaluated with the expression of ATOH1 and KI67 (***B***, ***C***). Animal numbers: *N* = 4 for pU6-*Notch1,2*-sh#1, *N* = 5 for pU6-*Notch1,2*-sh#2. Scale bars: 80 and 30 μm (***A***). ***D–K***, Electroporation with HES1 overexpression (OE) and KD vectors plus pCAG-H2B-GFP to the EGL. The rate of differentiation in electroporated cells was analyzed by immunohistochemistry with ATOH1 and KI67 (***E***, ***F***, ***J***, ***K***). The pCAG-empty vector (***D–F***) and the scrambled shRNA vector (Scramble) were used as controls (***I–K***). KD vectors for *Hes1* were checked the efficiency *in vitro* (***G***, ***H***). CAG-HES1-fusion-GFP were co-transfected with *Hes1* KD vectors to Neuro2a cells and the protein level were checked by Western blotting with GFP antibody (***G***, ***H***). β-Actin was used as a reference. Animal numbers: *N* = 5 for (***D–F***), *N* = 4 for (***I–K***). Sample numbers: *N* = 3 for (***G***, ***H***). Scale bars: 30 μm (***D***, ***I***). Data are shown as mean ± SEM; *p < 0.05, **p < 0.01, ***p < 0.001, Student’s *t* test.

**Figure 7.**
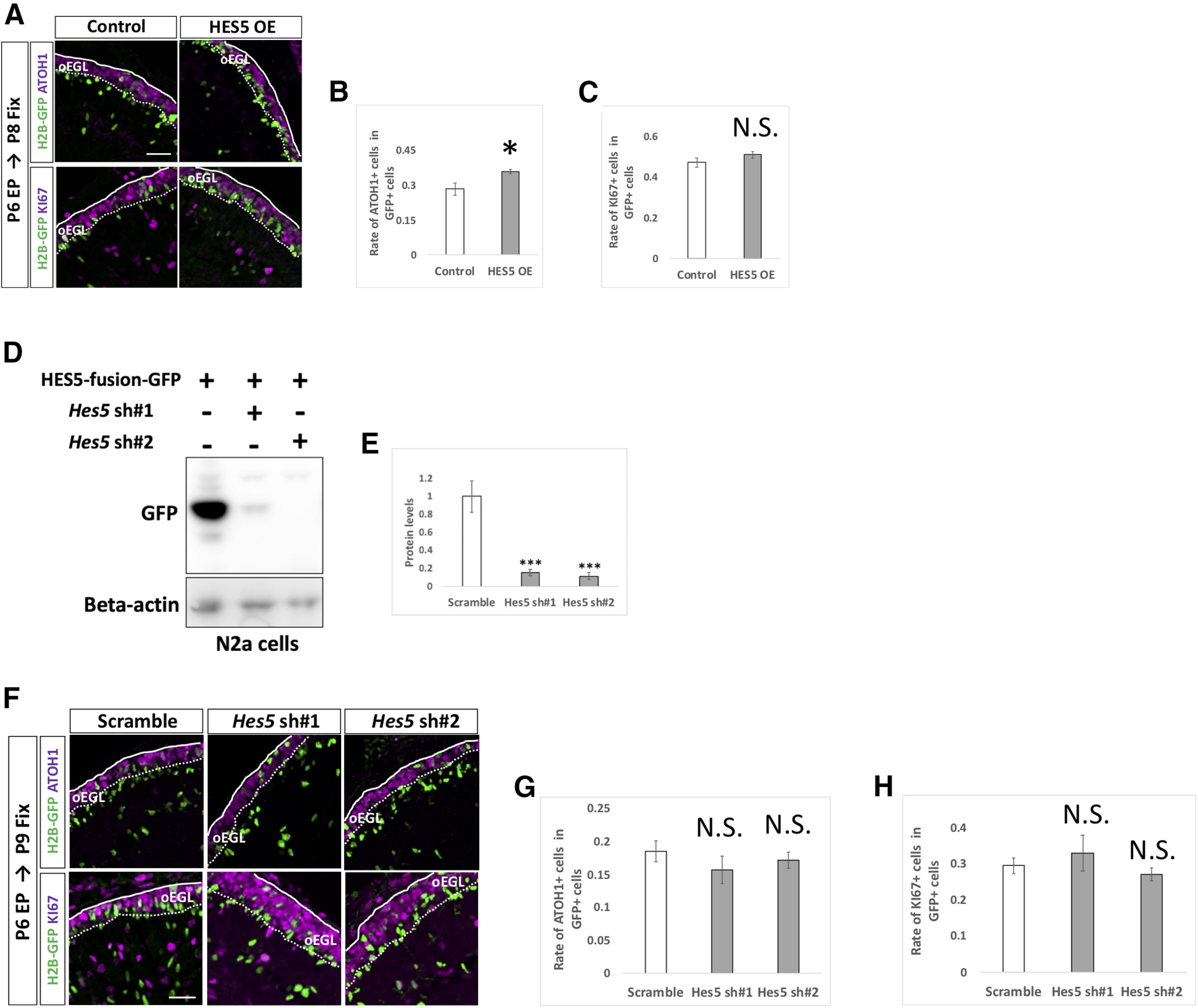
Endogenous HES5 is not physiologically involved in the differentiation of GCPs. ***A–H***, Overexpression (***A–C***) and KD (***F–H***) vectors for HES5 plus pCAG-H2B-GFP were electroporated to P6 cerebella, followed by fixation at indicated stages. Samples were immunostained with indicated antibodies and analyzed as in Figure 6*E*,*F*,*J*,*K*. The pCAG-empty vector (***A–C***) and Scramble (***F–H***) were used as controls. KD vectors for *Hes5* were checked the efficiency *in vitro* (***D***, ***E***). CAG-HES5-fusion-GFP were co-transfected with *Hes5* KD vectors to Neuro2a cells and the protein level were checked by Western blotting with GFP antibody (***D***, ***E***). β-Actin was used as a reference. Animal numbers: *N* = 5 for pCAG-HES5 OE, *N* = 4 for pU6-*Hes5*-sh. Sample numbers: *N* = 3 for (***D***, ***E***). Scale bars: 30 μm (***A***, ***F***). Data are shown as mean ± SEM; *p < 0.05, Student’s *t* test.

By this method, we introduced KD vectors for *Notch1* and *Notch2* plus pCAG-H2B-GFP into the P6 cerebella and analyzed them at P9 by immunostaining with KI67 and ATOH1, markers for GCPs (Fig. 6*A–C*). Interestingly, KD for *Notch2* significantly reduced the rates of ATOH1-positive cells as well as KI67-positive cells, while KD for *Notch1* did not show significant effects (Fig. 6*B*,*C*). This suggests that *Notch2* but not *Notch1* is involved in suppressing differentiation of proliferative GCPs to postmitotic GCs putatively in a cell autonomous manner. Next, we electroporated an overexpression vector (Fig. 6D*–F*) or KD vectors (Fig. 6*G–K*) for *Hes1* plus pCAG-H2B-GFP into the P6 cerebella, which were fixed at P8 and P9, respectively. While overexpression of HES1 increased ATOH1-positive and KI67-positive cells, KD of *Hes1* decreased those cells, implicating that HES1 suppresses differentiation from GCPs to GCs presumably in a cell autonomous manner. On the other hand, introduction of KD vectors for *Hes5* did not affect the ATOH1 and KI67 positivity of GCPs (Fig. 7*D–H*). This suggests that HES5 may not be involved in suppression of GCP differentiation into GCs, consistent with our finding that *Hes5* expression was very low compared with *Hes1* in GCP-like cells (Fig. 1*C*). Interestingly, introduction of a HES5 overexpression vector succeeded in suppressing differentiation of GCPs (Fig. 7*A–C*), similar to the effect of HES1. This led us to believe that, if overexpressed, HES5 has the ability to suppress GCP differentiation, probably via the same downstream pathway as that of HES1. However, as the expression of HES5 in GCPs is very low, endogenous HES5 is unlikely to be involved in that process. Altogether, these findings suggest that Notch2-dependent and Hes1-dependent Notch signaling is involved in maintaining GCPs in a proliferative and immature state in the developing cerebellum.

### JAG1 cell non-autonomously upregulates Notch signaling in surrounding GCPs

**Figure 8.**
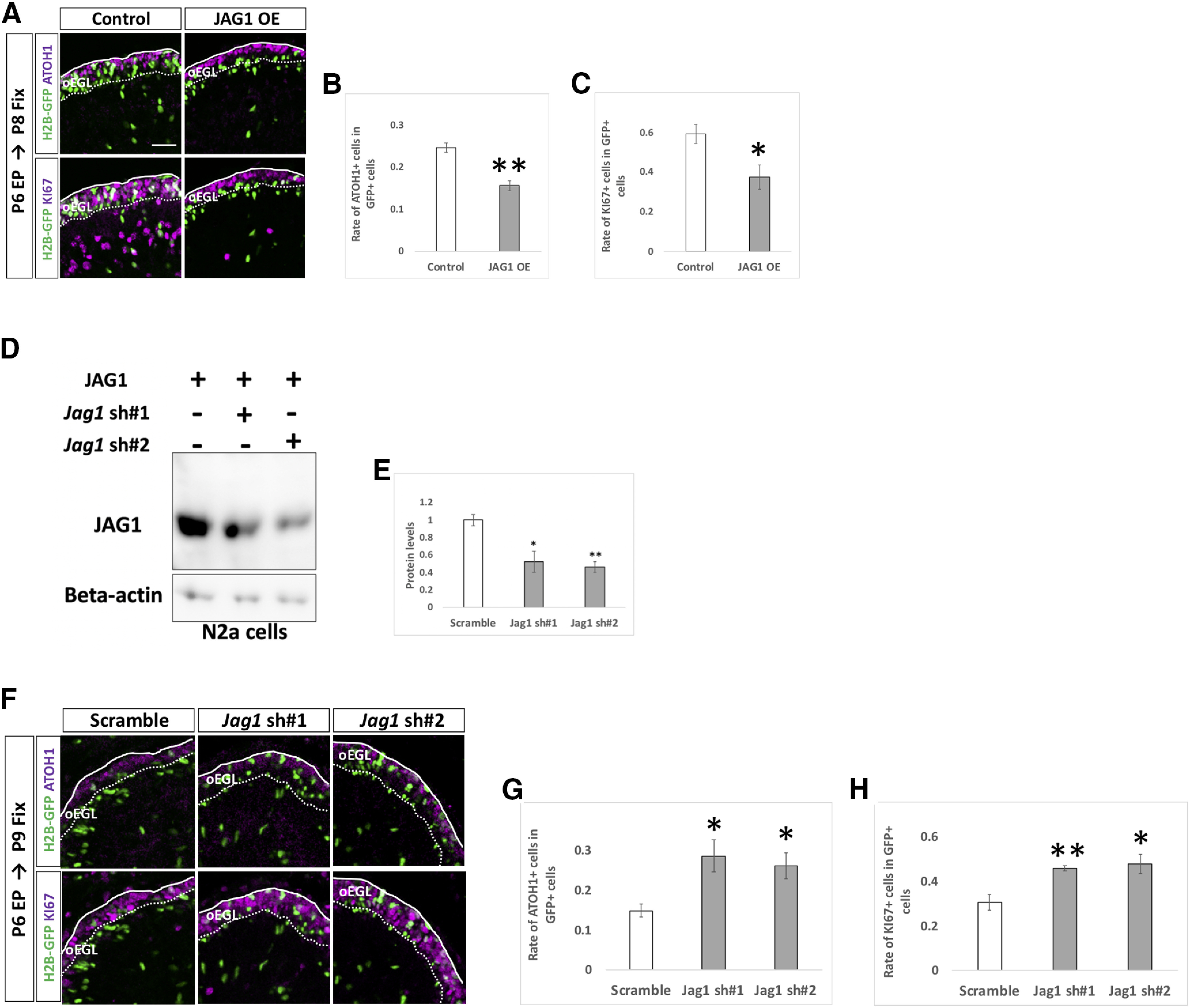
JAG1 cell autonomously promotes differentiation of GCPs. ***A–H***, Electroporation with overexpression (***A–C***) and KD (***F–H***) vectors for JAG1 plus pCAG-H2B-GFP into the P6 EGL. Electroporated cells were estimated by immunostaining for ATOH1 and KI67 at indicated developmental stages. The pCAG-empty vector (***A–C***) and Scramble (***F–H***) were used as controls. KD vectors for *Jag1* were checked the efficiency *in vitro* (***D***, ***E***). CAG-JAG1 were co-transfected with *Jag1* KD vectors to Neuro2a cells and the protein level were checked by Western blotting with JAG1 antibody (***D***, ***E***). β-Actin was used as a reference. Animal numbers: *N* = 4 for pCAG-JAG1 OE, pU6-*Jag1*-sh#1, *n* = 5 for pU6-*Jag1*-sh#2. Sample numbers: *N* = 3 for (***D***, ***E***). Scale bars: 30 μm (***A***, ***F***). Data are shown as mean ± SEM; *p < 0.05, **p < 0.01, Student’s *t* test.

Among the ligands for Notch signaling, we found that *Jag1* was strongly expressed in GCP-like cells compared with *Jag2*, and *Dll1,3,4* (Fig. 1*C*) and in the EGL (Fig. 1*G*). Therefore, we performed overexpression and KD experiments for *Jag1* with the same experimental strategy used for Notch and Hes genes (Fig. 6). While overexpression of JAG1 decreased rates of ATOH1 and KI67 positivity in the transfected cells (Fig. 8*A–C*), KD of *Jag1* led to the opposite results (Fig. 8*D–H*). This suggests that *Jag1* accelerates differentiation of Jag1-expressing GCPs into GCs, thus exhibiting contrasting effects to those of *Notch2* and *Hes1*.

**Figure 9.**
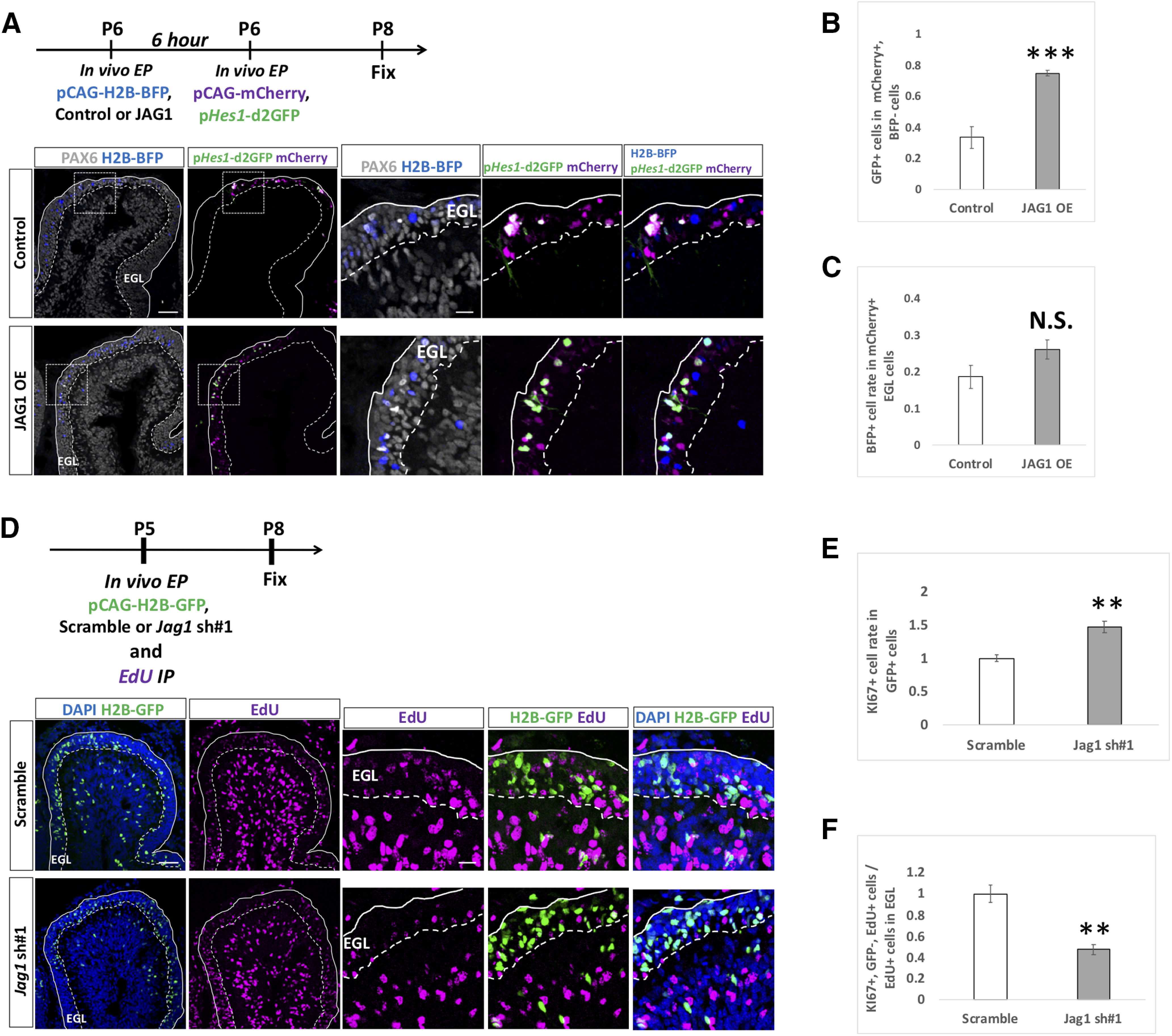
JAG1 cell non-autonomously upregulates Notch-signaling and suppresses differentiation of surrounding cells. ***A–C***, Double electroporations with a 6-h interval were performed to P6 EGL according to the indicated schedule. Rate of GFP+ cells in mCherry+ cells was drastically increased in JAG1-overexpressed (OE) mice compared with control (***A***, ***B***). Rates (20–30%) of the co-electroporated cells (BFP+ cells in mCherry+ cells) were not significantly different in control and JAG1 OE (***A***, ***C***). BFP-double positive and mCherry-double positive co-electroporated cells were excluded in the analysis of Figure 5*B*. Animal numbers: *N* = 4. Scale bars: 80 and 30 μm (***A***). ***D–F***, Electroporation with indicated vectors and intraperitoneal administration of EdU were performed in P5 mice, followed by fixation at P8. GFP+ cells were Jag1-downregulated cells and EdU+ GFP– cells were presumed neighboring cells. In the *Jag1* KD mice, the rate of GCPs in the signal sending cells were increased (***D***, ***E***). In contrast, differentiation of GCPs was accelerated in *Jag1* KD mice compared with control mice (***D***, ***F***). Animal numbers: *N* = 4. Scale bars: 80 and 30 μm (***D***). Data are shown as mean ± SEM; **p < 0.01, ***p < 0.001, Student’s *t* test.

It is known that, in many tissues, JAG1 acts as a ligand for Notch receptor proteins to increase Notch signaling in surrounding cells (Gomi et al., 2016). In addition, we showed that Notch signaling activity can be monitored by p*Hes1*-d2GFP in GCPs (Figs. 2*A*,*G*, 3*A*,*C*). Therefore, to test whether JAG1 increases Notch-signaling activity in surrounding cells, we performed a “double electroporation” experiment. We first electroporated JAG1 overexpression or control vector plus pCAG-H2B-BFP (nuclear localizing BFP) to P6 cerebella and performed the second electroporation with p*Hes1*-d2GFP plus pCAG-mCherry to the same cerebella at 6 h after the first electroporation. The animals were killed, and cerebella were fixed at P8. Under this experimental condition, some cells were singly transfected by the first or the second electroporation and some were doubly labeled by the both electroporations (Fig. 9*A*). Among cells transfected by the second electroporation (mCherry+ cells), the rates of doubly electroporated cells (BFP-positive cells) were not significantly different between the control and JAG1 introduced samples (Fig. 9*C*). We quantified GFP-positive cells in mCherry+ cells that had been transfected in the second electroporation but not in the first electroporation (BFP-negative). Interestingly, GFP-positivity among those cells was increased when JAG1 was introduced at the first electroporation (Fig. 9*A*,*B*). This suggests that the JAG1 expression in GCPs increases Notch-signaling activity of surrounding GCPs.

**Figure 10.**
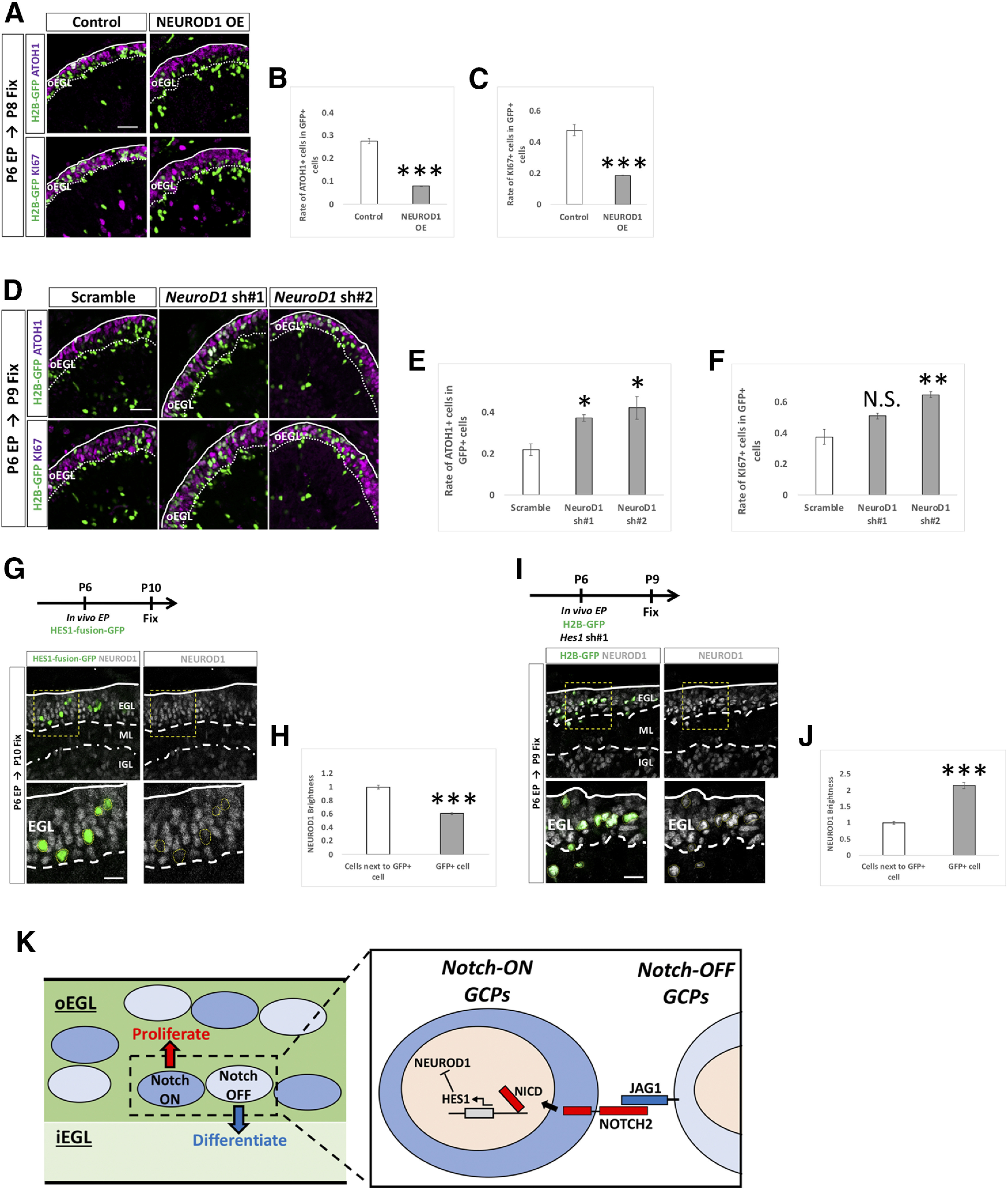
HES1 downregulates NEUROD1 in GCPs/GCs lineage. ***A–F***, Overexpression and KD vectors plus pCAG-H2B-GFP were electroporated to P6 cerebella, followed by fixation at indicated stages. Samples were immunostained with indicated antibodies and analyzed as in Figure 3E,*F*,*H*,*I*. Animal numbers: *n* = 4 for pCAG-NEUROD1 OE, pU6-*NeuroD1*-sh#1,2. Scale bars: 30 μm (***A***, ***D***). Data are shown as mean ± SEM; *p < 0.05, **p < 0.01, ***p < 0.001, Student’s *t* test. ***G***, ***H***, HES1-fusion-GFP was electroporated into P6 EGL and cerebella fixed at P10 (***G***). The fluorescence intensities for NEUROD1 were estimated in transfected (GFP-positive) cells (***H***). Animal numbers: *N* = 4. Scale bars: 30 and 10 μm (***A***). ***I***, ***J***, *Hes1* KD and pCAG-H2B-GFP were co-electroporated into P6 EGL, followed by immunostaining at P9 (***I***). Immunofluorescence intensities for NEUROD1 were estimated (***I***, ***J***). Animal numbers: *N* = 4. Scale bars: 30 and 10 μm (***A***). Data are shown as mean ± SEM; ***p < 0.001, Student’s *t* test. ***K***, A schematic model for Notch signaling in GCPs. In the oEGL of the developing cerebellum, there are two types of GCPs, Notch-signaling ON (signal receiving) and OFF (signal sending) GCPs. JAG1 expressed on the signal-sending GCPs interacts with NOTCH2 on the signal-receiving GCPs. This interaction induces cleavage of NICD by a γ secretase to upregulate *Hes1* expression in the signal-receiving GCPs. HES1 suppresses expression of NEUROD1, eventually maintaining GCPs at immature and proliferative status. Signal-sending GCPs express only a small amount of HES, resulting in expression of NEUROD1 and subsequent differentiation into GCs.

Next, to examine the cell non-autonomous effect by KD for *Jag1*, we designed an experiment that combined electroporation and EdU-incorporation techniques. We electroporated the KD vector for *Jag1* plus pCAG-H2B-GFP in GCPs of P5 mice with a simultaneous intraperitoneal injection of EdU and then fixed the samples at P8 for immunostaining (Fig. 9*D*). In this experiment, EdU-positive/GFP-negative cells could be regarded as progeny cells of proliferative GCPs that had not been introduced with the *Jag1* KD vector at P5. Furthermore, because GFP-positive cells were observed very densely in the electroporated EGL area (Fig. 9*D*), most or many of EdU-positive/GFP-negative cells in the electroporated area are likely offspring cells of non-transfected GCPs that had a contact with *Jag1* KD GCPs. As observed in Figure 8*F–H*, KI67-positive cells were also increased in *Jag1* KD cells (GFP-positive cells) in this experiment (Fig. 9*D*,*E*). In contrast, the rate of KI67-positive cells was decreased in EdU-positive/GFP-negative cells (Fig. 9*D*,*F*). This implies that *Jag1* KD promotes differentiation of surrounding cells. Furthermore, together with the findings that JAG1 overexpression enhances Notch activity of surrounding cells (Fig. 9*A–C*) and that Notch2-Hes1-dependent Notch signaling keeps GCPs in an immature and proliferative state, this suggests that JAG1-expressing GCPs may maintain surrounding GCPs in a proliferative state via NOTCH2-HES1 pathway-dependent Notch signaling (Fig. 10*K*).

### Hes1 is involved in downregulating NEUROD1 expression

It has been suggested that the transcription factor, NEUROD1, is required for differentiation of GCs in the developing cerebellum (Pan et al., 2009). Consistent with this, in our experimental conditions, overexpression of NEUROD1 significantly decreased ATOH1-positive cells and KI67-positive cells (Fig. 10*A–C*). Introduction of KD vectors for *NeuroD1* caused the opposite results to the overexpression experiment (Fig. 10*D–F*). These observations confirmed that NEUROD1 is involved in promoting the differentiation of GCPs to GCs. To investigate the relationship between Notch signaling and NEUROD1 expression, we electroporated pCAG-HES1-fusion-GFP (HES1-GFP fusion protein) into GCPs at P6 and fixed the cerebella at P10. HES1-GFP overexpression reduced the immunofluorescence signals for NEUROD1 in the EGL (Fig. 10*G*,*H*). In contrast, *Hes1* KD displayed the opposite results (Fig. 10*I*,*J*). These observations suggest that Hes1 maintains the proliferative and immature states of GCs via regulation of NEUROD1.

## Discussion

During cerebellar development, numerous GCPs, packed in the oEGL, seem to constitute a uniform population. However, the molecular regulatory machinery underlying how maternal GCPs produce proportional numbers of sister GCPs and GCs has not been well understood. In this study, we found that there are two populations of GCPs in the oEGL, Notch-signaling ON and OFF cells. The former possess more immature and proliferative characteristics, while the latter have more differentiative and less proliferative features. Expression of JAG1 in GCPs affects surrounding cells to become Notch-signaling ON cells via the Notch2-Hes1 pathway. HES1 decreases NEUROD1 expression in the ON-GCPs that eventually differentiate into GCs.

Several studies have reported that Notch-related transcripts and proteins are expressed in the postnatal cerebellum by means of RT-PCR (Solecki et al., 2001), *in situ* hybridization (Tanaka et al., 1999; Irvin et al., 2001, 2004; Solecki et al., 2001; Stump et al., 2002; Eiraku et al., 2005), and immunostaining (Tanaka and Marunouchi, 2003). However, some of the data from these studies were contradictory. This might be caused by differences of experimental conditions and/or differences in the cell population purity. In this study, we aimed to distinctly investigate the gene expression in GCPs and GCs with the hypothesis that Notch-related molecules might be differentially expressed between the two cell types. We successfully purified GCP and GC-like cells (Kutscher et al., 2020). By quantitative RT-PCR analyses as well as immunohistochemistry, our results suggest that *Notch1*, *Notch2*, *Hes1*, and *Jag1* were prominently expressed in GCPs, while *Hes5*, *Jag2*, and *Dll1* were significantly expressed in GCs. This differential expression between GCPs and GCs seems to partly explain the previous contradictory findings.

Several studies have previously reported phenotypes of knock-out (KO) mice for Notch-related genes (Eiraku et al., 2005; Weller et al., 2006; Komine et al., 2007; Hiraoka et al., 2013) during postnatal cerebellar development. Conditional KO (cKO) of *Notch1*, *Notch2*, *RBPJ*, or *Dll* in astroglial cell lineage with a GFAP-Cre line resulted in disorganized positioning and morphology of Bergmann glia (BG; Komine et al., 2007; Hiraoka et al., 2013), although GCP/GC-related phenotypes were not assessed in those reports. In cKO mice for *Jag1* in the whole cerebellum, generated by crossing with an En2-Cre line, the position and morphology of BGs were also impaired. In those mice, the EGL was abnormally retained until P20, because of the aberrant GC migration (Weller et al., 2006). However, since *Jag1* is expressed in both BGs and GCPs/GCs, it was unclear whether *Jag1* expression in BGs or GCPs/GCs were responsible for the phenotype. In this study, by *in vivo* electroporation KD experiments, we clearly showed that *Jag1* expression in GCPs is involved in GCP differentiation. Another group analyzed conventional KO mice for *Dner*, a non-canonical ligand for Notch signaling, expressed in Purkinje cells (PCs) as well as GCs in the iEGL (Eiraku et al., 2002, 2005). In the mutant cerebellum, localization and morphology of BGs are impaired, while GC migration was delayed. The authors suggest that Notch signaling via DNER on PCs and NOTCH1 on BGs might be involved in BG differentiation, although there still remains the possibility that DNER expressed in GCs are responsible for the phenotype. In another *in vitro* cell and explant culturing study, it was reported that overexpression of NOTCH2 and HES1 suppressed process extension of GCs (Solecki et al., 2001). Although this data showed the ability of these molecules to affect GC differentiation, their physiological requirement remained unknown because of the lack of loss of function experiments.

Despite these previous reports on Notch signaling molecules, Notch activity has not been detected in postnatal cerebellar development. By monitoring promoter activities, we found the presence of Hes1-dependent, but not Hes5-dependent, Notch signaling activity in GCPs of the oEGL. This is the first report to directly show the presence of Notch signaling in the cerebellar EGL. Around half of GCPs are Notch-ON GCPs, while the others are Notch-OFF GCPs. *In silico* analyses suggested that the former were more immature and proliferative, while the latter possessed opposite features. KD and overexpression experiments by electroporation showed that NOTCH2 and HES1 are cell-autonomously required for Notch activity in GCPs. On the other hand, JAG1 cell non-autonomously upregulates the Notch activities of surrounding GCPs. Despite its expression in GCPs, NOTCH1 was not involved in the Hes1-dependent Notch signaling activity. It might be possible that NOTCH1 activates different downstream Hes-family genes, such as Hey.

In early neural development, *Hes1* is known to maintain stemness of mouse neural progenitor cells (NPCs) by suppressing the expression of proneural genes, such as *Ascl1* (Achaete-scute family bHLH transcription factor 1) and *Ngn2* (Neurogenin 2; Kageyama et al., 2007). In mouse ventral telencephalon NPCs, there is oscillating expression of bHLH transcriptional genes *Hes1*, *Ascl1*, and *Olig2* (Imayoshi et al., 2013). Once the fluctuation is lost and the expression of the genes are sustained, cell fates are determined to be astrocytes, neurons or oligodendrocytes (Imayoshi et al., 2013). This oscillation system may enable creation of minor differences within a uniform cell population, and eventually generate plural cell fates. Although we do not have any direct evidence, it is possible that Notch signaling also fluctuates among cerebellar GCPs during development. However, even if this was the case, the transition from ON to OFF or OFF to ON might occur very quickly, as we can barely detect the transient state GCPs, or intermediate promoter activities for *Hes1* as visualized by p*Hes1*-d2GFP (Fig. 3*D*,*E*). In addition, since *Hes1* promoter activities were not detected in mature GCs in the IGL, Notch signaling is thought to be fixed in the OFF state after GC differentiation.

The SHH subgroup of medulloblastoma, one of the major pediatric brain tumors, is known to be derived from the GC lineage, that is, GCPs/GCs (Goodrich et al., 1997). The Eberhart group previously showed that expression of *Notch2* but not *Notch1* was upregulated in medulloblastoma compared with normal pediatric cerebella, although they did not discriminate between SHH and the other subgroups (Fan et al., 2004). They also showed that *Notch2* and *Hes1* are involved in the proliferation of medulloblastoma-derived cell line. These observations, at least in part, seem to be consistent with our finding that the Jag1-Notch2-Hes1 pathway maintains GCPs in a proliferative state by upregulating Notch signaling. Therefore, this study may provide clues to understanding the mechanisms underlying tumorigenesis or growth of medulloblastoma and to finding potential therapeutic vulnerabilities.

In this study, we first visualized Notch-signaling in GCPs. NOTCH2 and HES1 are involved in Notch-signaling to maintain GCPs in an immature and proliferative state in a cell autonomous manner. This system may generate two distinct types of GCPs, NOTCH-ON and OFF, and contribute to production of an appropriate balance of sister GCPs and GCs from mother GCPs, eventually leading to the formation of the normal cerebellum. We believe that this study gives insights into understanding the basic machinery to produce different cell types from a seemingly uniform cell population in normal cerebellar development and also the pathology of medulloblastoma.

## Synthesis

Reviewing Editor: Christian Hansel, University of Chicago Decisions are customarily a result of the Reviewing Editor and the peer reviewers coming together and discussing their recommendations until a consensus is reached. When revisions are invited, a fact-based synthesis statement explaining their decision and outlining what is needed to prepare a revision will be listed below. The following reviewer(s) agreed to reveal their identity: NONE. Note: If this manuscript was transferred from JNeurosci and a decision was made to accept the manuscript without peer review, a brief statement to this effect will instead be what is listed below.

In this work, the authors identify two populations of cerebellar granule cell precursors (GCPs) in the outer external granular layer (oEGL), one with an active Notch-/Hes1 signaling and on with this signaling pathway inactive. They also show that JAG1 has a role to activate in a cell-non-autonomous way this pathway. They use a combination of in vivo knock-down and over-expression in the GCPs by in vivo electroporation.  There are only few major concerns regarding possible weak points of the paper, which however do not seem to affect its main conclusions.  MAJOR CONCERNS  Fig. 2: Hes1 doesn’t seem to be active in nearly as many cells as those which express NOTCH1, 2 or JAG1. A quantification of the relative number of d2GFP+ / mCherry+ cells could help to estimate the activation of Hes1 (as in panel F). The authors could also consider if they want to make their point even stronger by repeating the experiment and quantify the frequency of NOTCH2+ cells in which they observe the expression of Hes1-driven d2GFP, of mCherry, relative to NOTCH2+ mCherry+ d2GFP-negative as an estimation of penetrance of NOTCH2-induced activation of Hes1, as well as d2GFP+ NOTCH2+ cells compared to d2GFP+ NOTCH2-negative as an estimation of specificity of NOTCH2-induced activation. In the experiment reported in Fig. 2C-D, it could be helpful to quantify the relative number of Venus+/ mCherry+ at 8h or later on fixed tissue. It would be appropriate to report also the number of animals in which the observations were made, and, to report quantitative data as the average among animals.  Fig. 3: the bimodal distribution shown in panels D-E is crucial in the paper and based on 4 animals. The absence of inter-animal variability may raise the concern that the distribution of GFP intensity may depend on inter-animal variability of GFP expression. The data could be reanalyzed as average among animals ({plus minus} SEM), considering to use larger bins/categories than in panel D for convenience (e.g. 0-5, 5-20, 20-25, 25-40). If the bimodal distribution will be still evident the presence of 2 distinct populations of CGP cells (“Hes1-ON” and “Hes1-OFF”) would be confirmed.   MINOR POINTS  Fig. 6G-H, Fig. 7C-D, Fig. 8D-E: the main text does not seem to refer to these panels. Every panel may need to be described in the text if included in the figures.  Fig. 7C-D: two different pairs of panels have these labels
